# Temporal stability and spatial patterns of genetic diversity in populations of the climate‐vulnerable fucoid *Scytothalia dorycarpa*

**DOI:** 10.1111/jpy.70156

**Published:** 2026-03-24

**Authors:** Jane M. Edgeloe, Melinda A. Coleman, Georgina V. Wood, Samuel Starko, Matt J. Nimbs, Jacqueline Batley, Thomas Wernberg

**Affiliations:** ^1^ School of Biological Sciences University of Western Australia Perth Western Australia Australia; ^2^ Oceans Institute University of Western Australia Perth Western Australia Australia; ^3^ Department of Primary Industries and Regional Development National Marine Science Centre Coffs Harbour New South Wales Australia; ^4^ College of Science and Engineering Flinders University Adelaide South Australia Australia; ^5^ School of Molecular and Life Sciences Curtin University Perth Western Australia Australia; ^6^ Norwegian Institute of Marine Research His Norway

**Keywords:** Australia, CO1, *cox*3, genetics, haplotype, macroalgae, *rbc*L, *trn*W‐1

## Abstract

Climate change is driving the loss of genetic diversity, potentially limiting species' capacity to adapt to environmental change. Detecting changes in genetic diversity requires replicated temporal data, which is lacking for most species. Here, we combined contemporary and historical specimens of the climate‐vulnerable fucoid *Scytothalia dorycarpa* to assess genetic diversity across ~2700 km of its geographic range. We analyzed four conserved organellar markers (*rbc*L, CO1, *cox*3, and *trn*W‐1) using newly collected specimens and herbarium material to reconstruct past diversity. *Scytothalia dorycarpa* is endemic to Australia and has experienced climate‐mediated declines over recent decades. We found stability in haplotype diversity over the 16‐year sampling period, as well as across additional historical herbarium collections (1800s, 1883, 1960). We identified several common contemporary and historical haplotypes across the sampled range, but diversity patterns varied between markers. Nonetheless, consistent trends emerged for certain populations, with high, unique haplotype diversity consistently present across all markers and timepoints in the Cape Naturaliste‐Leeuwin region (Western Australia). Notably, both contemporary and historical (now extinct) warm‐edge populations had unique haplotypes that were absent elsewhere in the sampled range. These results demonstrate strong temporal stability in *S. dorycarpa* genetic diversity, with limited haplotype turnover, highlighting the resilience of sampled populations. The presence of unique haplotypes in specific populations underscores their role as reservoirs of evolutionary potential. By documenting long‐term stability alongside localized diversity losses, this study provides a critical baseline for understanding the processes shaping genetic variation in *S. dorycarpa* and predicting its responses to future climate change.

**Abbreviations**
CO1: partial cytochrome c oxidase subunit 1, mtDNA *cox*1‐5′ barcoding region
*cox*3: partial cytochrome oxidase subunit 3, mtDNA gene region
cpDNA: chloroplast DNA
CTAB: cetyltrimethylammonium bromide
*H*
: number of haplotypes
*H*_d_
: haplotype diversity
*K*
: average number of nucleotide differences
MHW: marine heatwave
mtDNA: mitochondrial DNA
PCR: polymerase chain reaction
*P*_i_
: nucleotide diversity
*rbc*L: Ribulose‐1,5‐bisphosphate carboxylase/oxygenase (RuBisCO) large subunit (rbcL) marker
RuBisCO: Ribulose‐1,5‐bisphosphate carboxylase/oxygenase
*S*
: number of segregating sites
TCS: Templeton‐Crandall‐Sing
*trn*W‐1: partial mtDNA intergenic spacer region

## INTRODUCTION

Genetic diversity is declining globally as a result of anthropogenic stressors (Hoban et al., 2022; Shaw et al., 2025), with mounting evidence of widespread losses across ecosystems, including marine environments (Coleman, Minne, et al., 2020; Gurgel et al., 2020; Lo Brutto et al., 2011; Provan, 2013; Starko et al., 2023). Under future climate scenarios, many marine species are predicted to experience range contractions and, in some cases, local or global extinctions, particularly in equatorward portions of their ranges (Martínez et al., 2018; Pinsky et al., 2020). Nonetheless, the long‐term persistence of species will likely depend on the extent of standing genetic variation, which affects their adaptive capacity to tolerate shifting environmental conditions (Hoffmann & Sgro, 2011; Waldvogel et al., 2020). Reconstructing historical genetic baselines is crucial for assessing the extent to which genetic diversity is being maintained or eroded. Such baselines provide essential context for evaluating population trajectories and for identifying populations that may be most resilient to future environmental change. Spatial patterns of genetic diversity can reveal signals of local adaptation, historic gene flow, and connectivity (López‐Goldar & Agrawal, 2021; Meek et al., 2023; Vranken et al., 2021), while temporal changes can provide insight into recent evolutionary responses to environmental change (Aitken & Whitlock, 2013; Palumbi et al., 2019). Harnessing this knowledge is essential for developing proactive, spatially informed management strategies that conserve evolutionary potential and safeguard the adaptive resilience of populations under both present and future climatic conditions (Breed et al., 2019; Coleman, Wood, et al., 2020; Wood et al., 2024).

To predict how populations will respond to changing environmental conditions, it is crucial to understand how genetic diversity is distributed between and within populations, particularly across the broad geographic ranges of species which typically span diverse environmental conditions (Waldvogel et al., 2020). One of the main processes shaping this distribution is gene flow, which acts across spatial scales and provides insight into the degree to which populations are isolated or interconnected (Hedgecock et al., 2007; Lowe & Allendorf, 2010). Beyond contemporary connectivity, patterns of genetic diversity in the marine realm also reflect the influence of historical seascape features such as past continental shelf margins (Falvey & Mutter, 1981; Falvey & Veevers, 1974), sea‐level changes (Bryant, 1992; Nimbs et al., 2023), and glacial cycles (i.e., Lewis et al., 2013). These features can shape diversity and structure by sustaining species expansions and providing new ecological opportunities for populations to establish or by isolating gene pools as portions of species ranges become fragmented (Hellberg, 2009; Pavlova et al., 2017; Templeton et al., 1990). Finer patterns can be explained by present or past ocean current dynamics (Coleman et al., 2013), including major boundary currents and upwelling zones, limiting and mediating transport of propagules from one source to another (Bashevkin et al., 2020; Coleman, Roughan, et al., 2011; Gaylord et al., 2000; Palardy & Witman, 2014). Across southern Australia, repeated glacial and interglacial cycles over the last several hundred thousand years have driven substantial changes in coastline extent, ocean temperatures, and the strength of boundary currents (Barrows et al., 2002; Cadd et al., 2021; Chang et al., 2015; Lewis et al., 2013). Cooler glacial periods likely enabled the broad expansion of cool‐temperate marine species across newly available habitats, whereas warmer interglacial phases may have reduced suitable thermal environments, leading to population contraction and fragmentation (Lewis et al., 2013). These long‐term climatic oscillations also altered dispersal pathways by influencing major current systems (Akhir et al., 2020), thereby shaping historical patterns of connectivity and isolation. Together, these spatial and historical processes leave genetic signatures that can be used to reconstruct past population dynamics, revealing how populations have responded to environmental and habitat disturbances over time.

Genetic diversity may be particularly important for sessile marine species, which have limited capacity to shift their distributions in response to environmental change and instead depend on their underlying genetic composition and phenotypic plasticity (i.e., the ability to alter physiology, morphology, or behavior) to persist (Donelson et al., 2019; King et al., 2018). Seaweeds are key sessile habitat‐forming species that exemplify this vulnerability, particularly in regions undergoing rapid climatic shifts (Bartsch et al., 2016; de la Hoz et al., 2019; Straub et al., 2016; Tempera et al., 2021; Wernberg et al., 2011). One of the largest temperate reef systems in the world, Australia's Great Southern Reef, encompasses some of the most diverse temperate seaweed ecosystems on earth (Bennett et al., 2016). Historically stable climate, geographic isolation, and oceanographic features that include large boundary currents are factors shaping the high levels of endemism and biodiversity of the system, particularly in seaweeds (Phillips, 2001; Wernberg et al., 2013). The drivers of genetic diversity and structure vary between seaweed species (Coleman, Chambers, et al., 2011; Gurgel et al., 2020; Macaya & Zuccarello, 2010; Mamo et al., 2021; Neiva et al., 2012; Vranken et al., 2021; Wood et al., 2021). Therefore, understanding spatial patterns of genetic diversity and structure is essential for identifying regions of high diversity, restricted connectivity, or unique evolutionary lineages. Moreover, for most species, it is generally not fully known to what extent genetic diversity and composition varies temporally (but see Coleman, Minne, et al., 2020; Coleman, Wood, et al., 2020; Gurgel et al., 2020; Reynes et al., 2024), and thus, more work is needed to close this knowledge gap, particularly for climate‐vulnerable species.

*Scytothalia dorycarpa* (Seirococcaceae, Fucales) is a habitat‐forming fucoid seaweed species that is endemic to the Great Southern Reef, inhabiting depths of 3–30 m across its ~3000 km range (Coleman & Wernberg, 2017; Smale & Wernberg, 2013). *Scytothalia dorycarpa* evolved during a period of cool, climatically stable conditions (Phillips, 2001) and is now observed across a relatively narrow range of ocean temperatures (i.e., summer temperatures varying from 18 to 24°C) and is sensitive to temperatures above its thermal range (Smale & Wernberg, 2013; Xiao et al., 2015). *Scytothalia dorycarpa* has a gametic life history similar to other fucoids, with a diploid adult producing haploid spermatozoids and eggs, which following fertilization develop directly into diploid juveniles (Andrews et al., 2014). In the austral summer of 2011, populations of *S. dorycarpa* in Western Australia were exposed to a marine heatwave (MHW), which resulted in a ~100‐km range contraction at the low‐latitude rear‐edge (Smale & Wernberg, 2013). In areas where *S. dorycarpa* persisted, there was little detectable change in percentage cover of *S. dorycarpa* from before (2008) compared to after (2012) the MHW (Gurgel et al., 2020), but there were declines in genetic (haplotype) diversity and identity. However, no knowledge exists on the nature of haplotype diversity and identity of the remaining populations of *S. dorycarpa* across the entirety of their geographic range or on now‐extinct populations and how these identities might naturally fluctuate over time.

Here, we assessed spatial and temporal haplotype diversity of *Scytothalia dorycarpa* across ~2700 km of its geographic range in western Australia and South Australia. We leveraged a rare opportunity to incorporate historical herbarium specimens and opportunistic sampling from various research programs to reconstruct changes in genetic composition across space and through time and to detect temporal shifts across 139 years but predominantly spanning a 16‐year period of more systematic sampling. We aimed to explore long‐term changes in haplotype identity and diversity both among regions and over time, providing critical insights into the stability or erosion of genetic diversity over time. In particular, we aimed to assess if there were any detectable spatial or temporal changes in haplotype diversity following the 2011 MHW, particularly at the warm range edge. Any loss of haplotype diversity, or of unique haplotypes, could have serious consequences for population persistence and adaptation. As such we aimed to use the historical baselines to estimate changes in possible adaptive potential across space and before and after the 2011 MHW.

## MATERIALS AND METHODS

### Contemporary sampling

**FIGURE 1:**
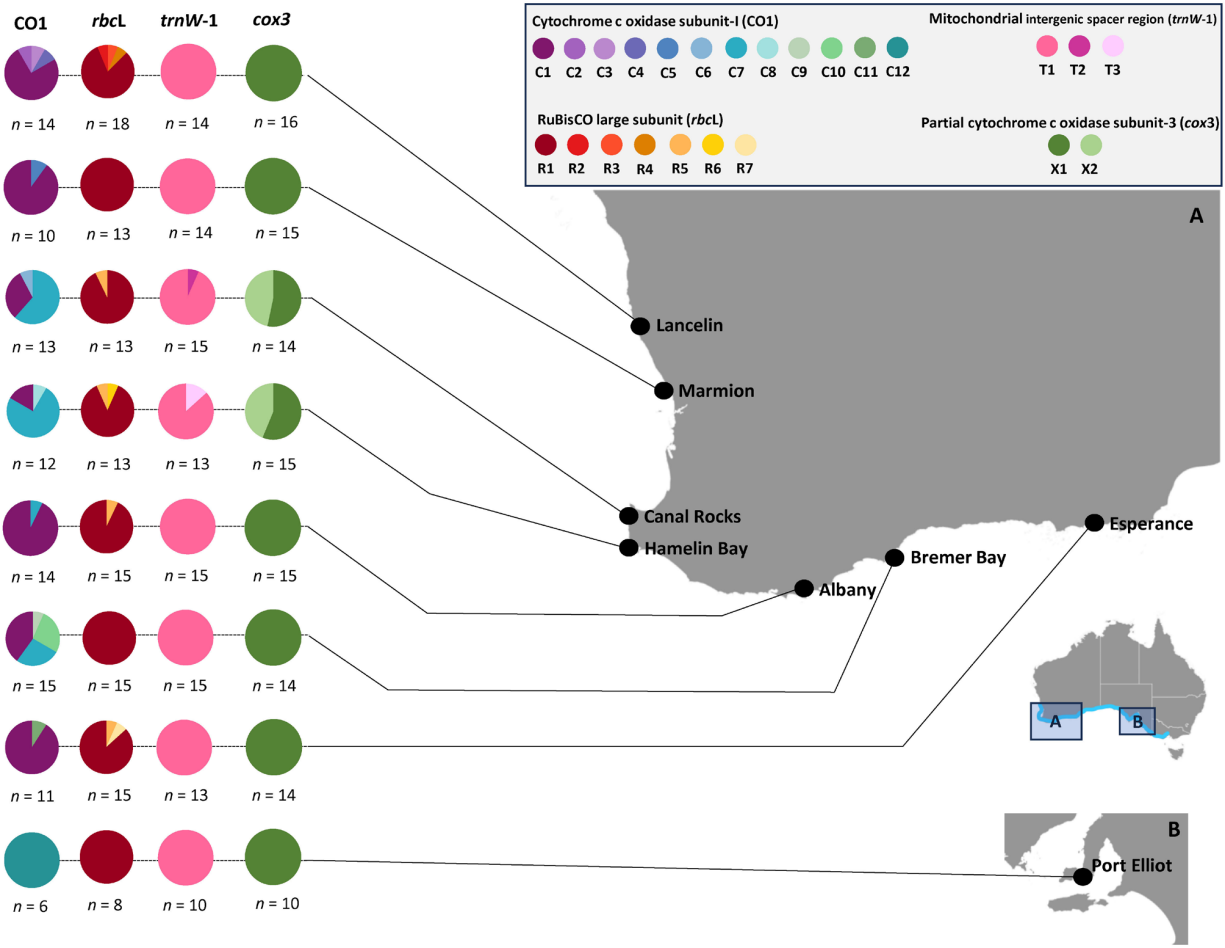
Haplotype identity distribution of *Scytothalia dorycarpa* at eight locations in Australia sampled in the year 2022. Haplotypes are based on four gene regions: cytochrome c oxidase subunit‐I (CO1) mitochondrial gene region, RuBisCO large chloroplast subunit (*rbc*L) gene region, partial mtDNA intergenic spacer region (*trn*W‐1), and cytochrome c oxidase subunit‐III (*cox*3) mitochondrial gene region. Colors represent different haplotypes, with proportions represented as pie charts and number of samples represented as *n* = X. Contemporary geographic range of *S. dorycarpa* is shaded in blue on inset map.

We sampled *Scytothalia dorycarpa* individuals at eight locations along ~400 km of latitude and ~2300 km of longitude across the southwest portion of its geographic range in Western Australia and a minor portion of the eastern range in South Australia in 2022 (Figure 1). These locations encompass an environmental gradient of ~5°C sea surface temperature, capturing potential genetic variation and population structure across most of the ranges of this species. Within each location, *n* = 30 whole individuals (including holdfast) were sampled haphazardly >2 m apart at 8–14 m depth using SCUBA. Samples from the warm, rear‐edge location, Lancelin, were collected as beach wrack (*n* = 22), as no samples were observed in situ at the sampled locations during *n* = 6 dives on SCUBA at different sites. Lancelin experiences a prevailing southward surface current; therefore, we are confident the beach drift samples were not from areas further south. Samples at the near cool, leading‐edge location Port Elliot (South Australia; *n* = 10) were also collected as beach cast wrack and stored in silica gel. A total of *n* = 3 clean, unfouled, and low epiphyte coverage apical fronds from the upper half of each plant were selected for genetic processing. Fronds were cleaned of epiphytes by gentle scraping using a sterile scalpel blade, rinsed in fresh water, and blotted dried, then snap‐frozen in liquid nitrogen, and stored at −80°C until DNA extractions were performed.

### Historical samples

We obtained historical samples from two sources: herbarium specimens (not treated in formalin) and frozen specimens previously collected by one of the authors (T. Wernberg; see Table S1). For the herbarium specimens, we attained a list of all *Scytothalia dorycarpa* specimens stored in Australian herbaria. From the list, we selected 19 samples from three of the regions sampled above and four populations from the northern range that are now extinct (Geraldton, Dongara, Cliff Head, and Jurien Bay; Table S2) for sequencing. Frozen samples included 46 *S. dorycarpa* specimens collected in Marmion (2006, *n* = 3), Jurien Bay (2008, *n* = 8), and Hamelin Bay (2008, *n* = 15 and 2010, *n* = 20) and stored in a −20°C freezer as whole individuals (Table S2). Samples were collected across 139 years (albeit with limited sample sizes for older specimens). At Marmion, collections were made 16 years apart (2006 and 2022). At Hamelin Bay, samples were collected at three time points: 2008, 2010, and 2022 (Table S2).

### Genetic sampling and gene amplification

DNA was extracted with the Qiagen DNeasy^®^ Plant Mini Kit (Qiagen, Germany) following a modified protocol (Vranken et al., 2021). DNA quantity, quality, and purity were assessed using the dsDNA high sensitivity Qubit^®^ Fluorometer (ThermoFisher Scientific, Australia) and stored at −20°C until further processing. Contemporary genomic DNA was amplified first and separately from historical DNA, as these samples had higher DNA concentrations and quality and could be used to confirm successful amplification of target gene regions. A second DNA extraction was performed on herbarium specimens using a cetyltrimethylammonium bromide (CTAB) protocol (Doyle & Jane, 1987). DNA that was >1 ng · μL^−1^ was diluted in MQ water at a 1:20 dilution factor to reduce inhibitor concentrations (Sidstedt et al., 2020). Diluted DNA was polymerase chain reaction (PCR) amplified in 25‐μL volumes using the Thermo Scientific™ Phire Plant Direct PCR Master Mix. Each 25‐μL PCR reaction consisted of 8 μL of H_2_O, 12.5 μL Phire Plant Direct PCR Master Mix, 1.25 μL of each of the forward and reverse primers, and 2 μL of 1:20 diluted DNA.

Due to the challenges inherent in extracting DNA from old herbarium specimens and the well‐known difficulty of obtaining high‐quality DNA from fucoids more generally, owing to their thick cell walls, abundant polysaccharides, polyphenolics, and other PCR‐inhibitory compounds (McCandless, 1981), we targeted the Ribulose‐1,5‐bisphosphate carboxylase/oxygenase (RuBisCO) large subunit (*rbc*L) cpDNA gene marker, and three mtDNA gene markers: the mtDNA partial cytochrome c oxidase subunit 1, *cox*1‐5′ barcoding region (CO1), partial co‐3 (*cox*3) mtDNA gene region, and partial mtDNA intergenic spacer region (*trn*W‐1; Table S1). These organellar markers have been widely applied to examine within‐species patterns of genetic diversity in brown seaweeds, with previous studies demonstrating variable levels of diversity in brown seaweeds across the target gene regions (Coleman et al., 2022; Durrant et al., 2015; Nimbs et al., 2023), with published sequences for *Scytothalia dorycarpa* in Gurgel et al. (2020) demonstrating evident differences in genetic diversity between two gene markers (*rbc*L and *cox*3) across a ~300 km latitudinal gradient.

DNA obtained from herbarium specimens were highly fragmented (<50–100 bp smear against ladder for raw DNA extract) and had a low concentration (<1 ng · μL^−1^) compared to the contemporary sampled and frozen samples. A PCR for *rbc*L and CO1 gene markers was attempted for all samples using the primers described in Table S1. Only three herbarium samples amplified for the CO1 gene with none amplifying for the *rbc*L gene; therefore, internal primers were designed for the *rbc*L and CO1 gene markers based on sequences generated from contemporary samples using Geneious Prime 11.1.5 (Biomatters Ltd., Auckland, New Zealand). Those samples that did not produce amplicons in the first round were then PCR amplified using three separate internal subsequences per marker (Table S1). To do this, three internal primer pairs were created for each gene marker (Table S1); PCR amplification produced three short‐read internal sequences that when concatenated formed one continuous usable marker sequence. For some samples, two to four PCR reactions with small adjustments to cycling reaction mix were performed until successful amplicons were produced for sequencing.

A gradient PCR was performed to determine the optimal annealing temperature of each primer pair (Table S1). The PCR cycling protocol followed those described in Nimbs et al. (2023), with an amendment of initial denaturation of 98°C for 3 min. A volume of 2 μL of unpurified PCR product was visualized using a 2% agarose gel to determine amplicon size (Table S1). All pre‐PCR work, including DNA extractions, reagent preparation, and PCR setup, was conducted on sterilized surfaces, with aerosol‐resistant filter tips and the routine inclusion of PCR negative controls to minimize and assess contamination. Unpurified PCR amplicons that produced a clear band on the gel at the marker‐appropriate bp interval and negative control were sent to the Australian Genomic Research Facility (AGRF), Perth, where dual‐direction sequencing was performed using Sanger Sequencing on an Applied Biosystems 3730xl capillary sequencer, using Big Dye Terminator (BDT) chemistry version 3.1 (Applied Biosystems) under standardized cycling PCR conditions. Per sequencing run, one technical replicate of the same DNA extract was included between runs to ensure consistency of sequences.

### Sequence analysis

Raw forward and reverse sequences (.abi files) were imported and assembled de novo using Geneious Prime^®^ version 2022.2.2 (Biomatters Ltd., Auckland, New Zealand). Sequences were quality controlled by inspecting automated base‐calls against chromatograms. Consensus sequences were aligned in Geneious Prime 11.1.5 (Biomatters Ltd., Auckland, New Zealand) using the MUSCLE algorithm and default settings (Edgar, 2004). The forward and reverse primer ends were trimmed to remove ambiguities and to ensure consistency across sequences. For quality control, the *rbc*L and *cox*3 gene sequences (including herbarium reads) were aligned to the published *Scytothalia dorycarpa* sequences in Gurgel et al. (2020) using the BLAST alignment tool (Wheeler & Bhagwat, 2007). As there were no published CO1 and *trn*W‐1 sequences for *Scytothalia*, the CO1 sequences were aligned to *Phyllospora comosa* sequences (Durrant et al., 2015), and the *trn*W‐1 sequences were aligned to *Sargassum natans* sequences in Geneious Prime to ensure the correct gene regions had been amplified. For historical sequences when independent replicate PCRs were not successful, haplotype calls were manually inspected and confirmed against chromatograms to ensure accuracy. The nucleotide alignments were exported from Geneious Prime for further analyses.

### Haplotype diversity analysis

Patterns of genetic (haplotype) diversity among *Scytothalia dorycarpa* samples across the sampling range were assessed using the DNA Sequence Polymorphism software (DNAsp) version 6.12.03 (Barcelona, Catalonia, Spain; Rozas et al., 2017). Contemporary sequence sets were defined according to the eight sample sites (Lancelin, Marmion, Canal Rocks, Hamelin Bay, Albany, Bremer Bay, Esperance, and Port Elliot), and historical sequence sets were defined according to the six regions (Geraldton, Jurien Bay, Lancelin, Marmion, Canal Rocks, and Hamelin Bay). Genetic diversity metrics were calculated for each population: *K*, average number of nucleotide differences; *S*, number of segregating sites; *H*, number of haplotypes; *H*_d_, haplotype diversity; and *P*_i_, nucleotide diversity. For visualization, a Templeton‐Crandall‐Sing (TCS) haplotype network (Clement et al., 2000) was reconstructed using the PopART software (Leigh et al., 2015).

Sequences from specimens successfully sequenced for all four gene regions were concatenated in Geneious Prime^®^ version 2022.2.2 (biomatters Ltd., Auckland, New Zealand) to form a single multi‐marker alignment, and a partition file (gene length) was created as a text file. The alignment was imported into W‐IG‐Tree builder (Trifinopoulos et al., 2016) and a best‐fit model was applied to each partition file using the ModelFinder algorithm (Kalyaanamoorthy et al., 2017). In this study, the algorithm determined the best‐fit models to be F81 + F, F81 + F, HKY + F and F81 + F for COI, *cox*3, *rbc*L and *trn*W‐1 partitions, respectively, with the resultant tree visualized using FigTree 1.4.4 (Figure S1; Rambaut & Drummond, 2012). The F81 + F model assumes equal transition and transversion rates, with the HKY + F model allowing for different rates for transitions and transversions.

## RESULTS

### Contemporary geographic patterns of haplotype diversity and identity

**FIGURE 2:**
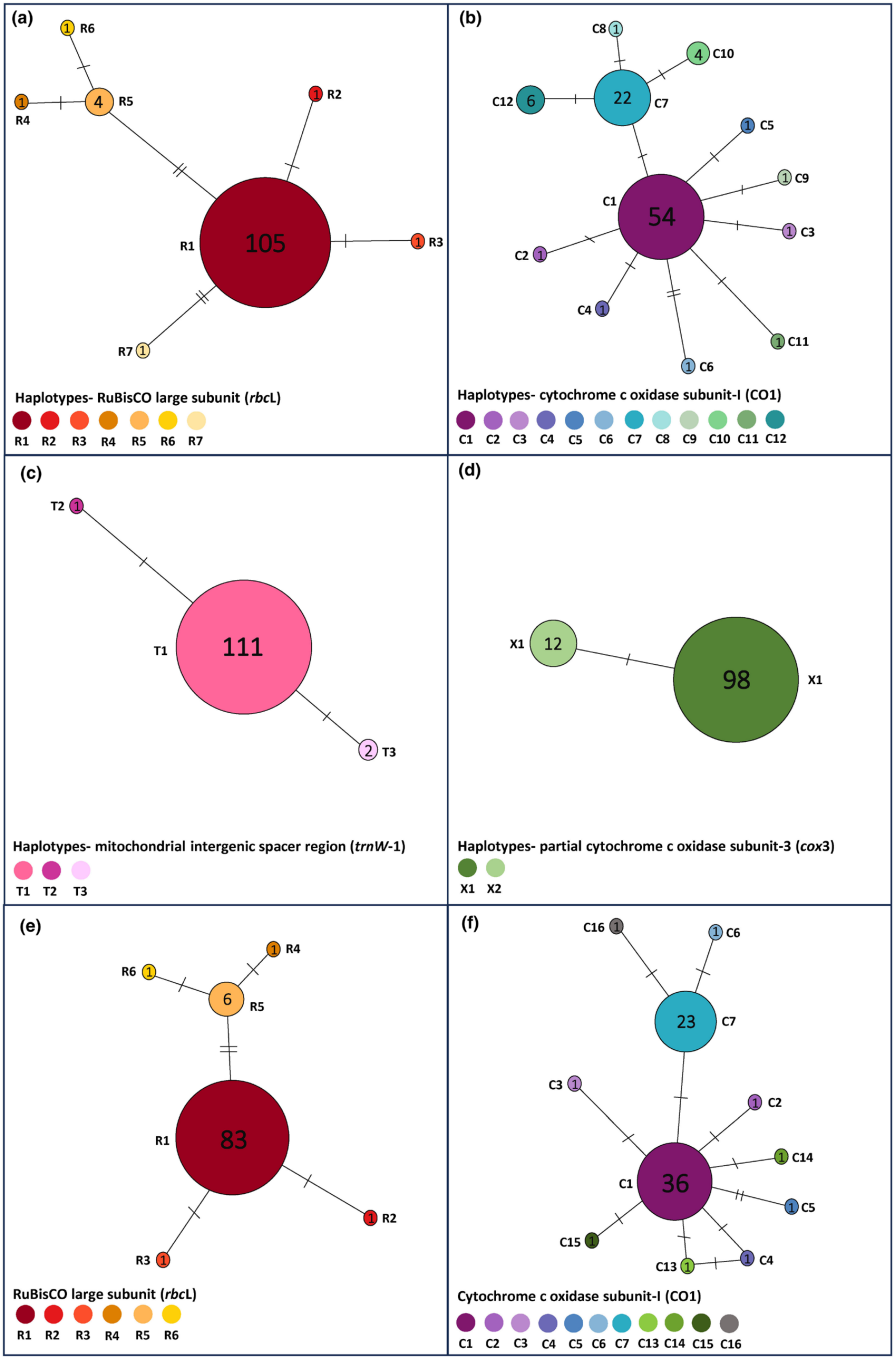
Templeton‐Crandall‐Sing (TCS) haplotype network of *Scytothalia dorycarpa* based on contemporary sequences (a–d) and historical sequences (e and f). Circle size is representative of sample size (with sample size provided inside circle) and network link hatch marks represent base pair substitutions. Haplotypes are listed next to node.

We generated a total of 94 CO1 (704 bp), 114 *rbc*L (902 bp), 114 *trn*W‐1 (152 bp), and 110 *cox*3 (140 bp) sequences from contemporary collections (year 2022) for this study (Table 1). Across these contemporary samples, patterns of haplotype diversity varied between markers, with a total of 12 haplotypes present for CO1 (C1–C12), seven for rbcL (R1–R7), three for *trn*W‐1 (T1–T3), and two for *cox*3 (X1–X2; Table 1; Figures 1 and 2). Shared, widely distributed, common haplotypes across the entire sampled area were observed for the *rbc*L (R1), *trn*W‐1 (T1), and *cox*3 markers (X1), with a dominant COI haplotype (C1) shared among all locations except the near‐leading edge location, Port Elliot, which composed a single, private haplotype (C11; Figures 1 and 2).

**TABLE 1:** Contemporary haplotype diversity metrics for *Scytothalia dorycarpa* at eight locations in Australia (2022) for the cytochrome c oxidase subunit‐1 (CO1) mtDNA region, RuBisCO large chloroplast subunit (*rbc*L) gene region, partial mtDNA intergenic spacer region (*trn*W‐1), and cytochrome c oxidase subunit‐III (*cox*3) mtDNA region.

Marker	Location	*n*	*K*	*S*	*P*_i_	*H*	*H*_d_
CO1	Lancelin	14	0.429	3	0.001	4	0.396
	Marmion	10	0.200	1	0.000	2	0.200
	Canal Rocks	13	0.821	3	0.001	3	0.564
	Hamelin Bay	11	0.509	2	0.001	3	0.473
	Albany	14	0.264	1	0.000	2	0.264
	Bremer Bay	15	1.086	3	0.002	4	0.743
	Esperance	11	0.182	0	0.000	2	0.182
	Port Elliot	6	0.000	0	0.000	1	0.000
	All locations	93	0.436	13	0.001	12	0.353
*rbc*L	Lancelin	18	0.556	5	0.001	4	0.314
	Marmion	13	0.000	0	0.000	1	0.000
	Canal Rocks	15	0.267	2	0.000	2	0.133
	Hamelin Bay	15	0.629	3	0.001	3	0.257
	Albany	15	0.267	2	0.000	2	0.133
	Bremer Bay	15	0.000	0	0.000	1	0.000
	Esperance	15	0.533	4	0.001	3	0.257
	Port Elliot	8	0.000	0	0.000	1	0.000
	All locations	114	0.282	16	0.000	7	0.137
*trn*W‐1	Lancelin	16	0.000	0	0.000	1	0.000
	Marmion	15	0.000	0	0.000	1	0.000
	Canal Rocks	14	0.143	1	0.001	2	0.143
	Hamelin Bay	15	0.233	1	0.002	2	0.233
	Albany	15	0.000	0	0.000	1	0.000
	Bremer Bay	14	0.000	0	0.000	1	0.000
	Esperance	15	0.000	0	0.000	1	0.000
	Port Elliot	10	0.000	0	0.000	1	0.000
	All locations	114	0.051	2	0.000	3	0.050
*cox*3	Lancelin	14	0.000	0	0.000	1	0.000
	Marmion	14	0.000	0	0.000	1	0.000
	Canal Rocks	15	0.533	1	0.004	2	0.533
	Hamelin Bay	13	0.527	1	0.004	2	0.527
	Albany	15	0.000	0	0.000	1	0.000
	Bremer Bay	15	0.000	0	0.000	1	0.000
	Esperance	13	0.000	0	0.000	1	0.000
	Port Elliot	10	0.000	0	0.000	1	0.000
	All locations	110	0.133	2	0.001	2	0.133

Abbreviations: *H*, number of haplotypes; *H*_d_, haplotype diversity; *K*, average number of nucleotide differences; *n*, sample size of DNA sequences in population set; *P*_i_, nucleotide diversity; S, number of segregating sites.

For the CO1 marker, there was local scale variation in patterns of haplotype diversity (Table 1; Figure 1). Across all markers, haplotype diversity was greatest in the Cape Naturaliste‐Leeuwin locations, Hamelin Bay (mean *H*_d_ = 0.373) and Canal Rocks (mean *H*_d_ = 0.343), with Hamelin Bay containing 10 different haplotypes across the four markers (Table 1; Figure 1). The warm‐edge location, Lancelin, had shared haplotypes with central locations but was somewhat genetically distinct as it contained private haplotypes for both the CO1 (C2, C3, and C4) and *rbc*L (R2, R3, and R4) markers (Figure 1). Notably, the vast majority of samples from Lancelin clustered within the large clade in the phylogenetic tree, indicating shared ancestry (Figure S1). The near‐leading edge location Port Elliot was genetically distinct from all other locations for the CO1 marker (C11); however, it had the common haplotypes (R1, X1, T1) for the other markers (Figure 1). Notably, Port Elliot also formed its own group on the phylogenetic tree, attributed to the unique CO1 haplotype (Figure S1).

Consistently across all four markers, Hamelin Bay and Canal Rocks contained haplotypes that were private to the Cape Naturalist‐Leeuwin region (Figures 1 and 2) and not observed in locations north or east. Notably, in the phylogenetic tree, samples from Canal Rocks and Hamelin Bay that harbored private haplotypes formed a distinct clade, indicating genetic divergence from the remaining populations (Figure S1). Interestingly, the near warm range edge population, Marmion, only contained a private haplotype for the CO1 marker (C5) but consistently lacked any variation in haplotype and nucleotide diversity across all other markers, sharing the common haplotypes across all markers (Table 1; Figure 1).

### Historical patterns of temporal haplotype diversity and identity

**FIGURE 3:**
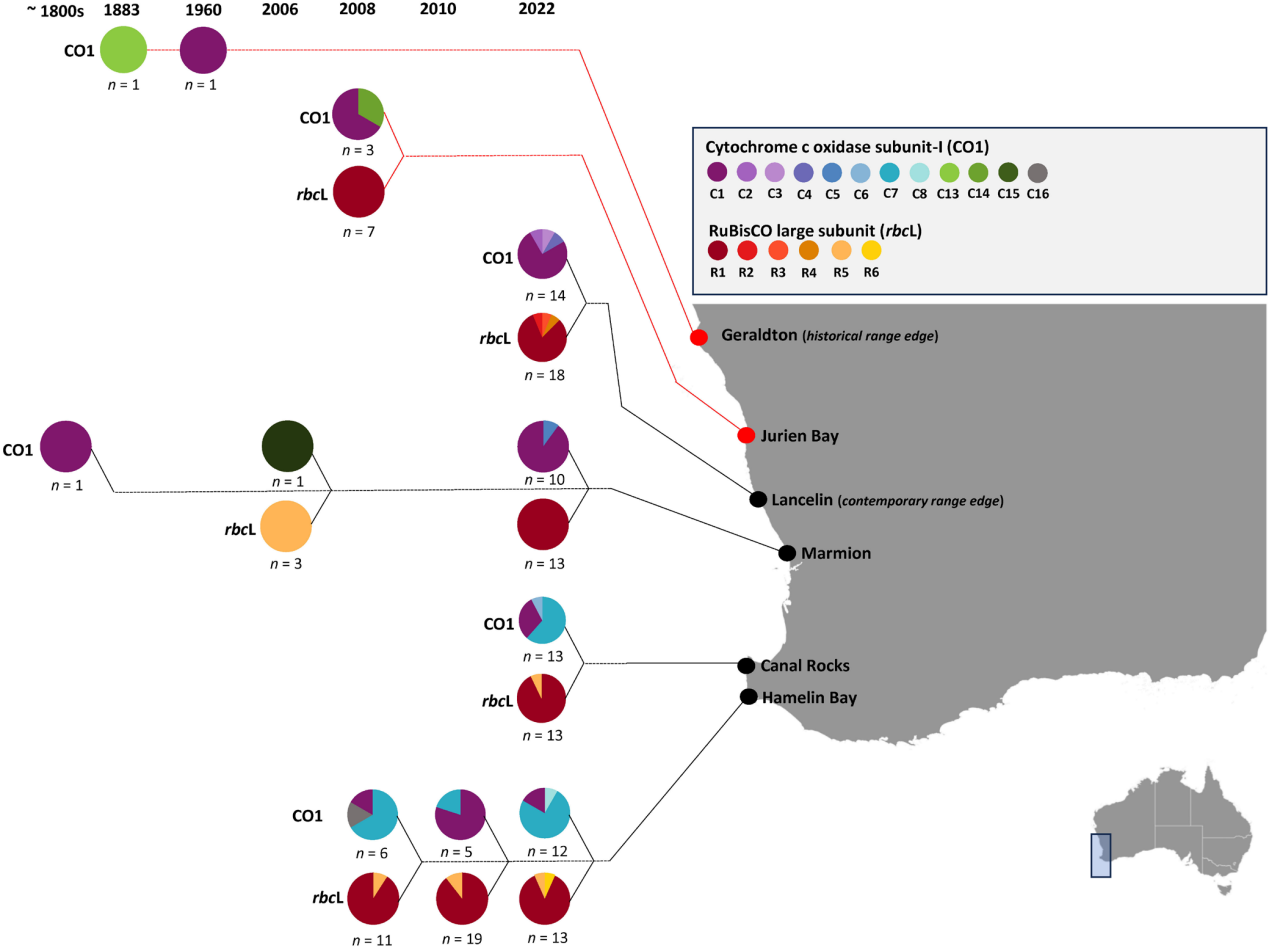
Haplotype identities of *Scytothalia dorycarpa* at six locations in Western Australia sampled between the years 1883 and 2022 from historical and contemporary specimens. Haplotypes are based on two gene regions: Cytochrome c oxidase subunit‐I (CO1) mitochondrial gene region and RuBisCO large chloroplast subunit (*rbc*L). Colors represent different haplotypes, with proportions represented as a pie chart and number of samples represented as *n* = X. Historical extinct locations denoted in red.

A total of 18 CO1 gene sequences (*n* = 3 herbarium samples, *n* = 15 historical samples) and 40 *rbc*L gene sequences (*n* = 40 historical samples) were successfully recovered from historical specimens, spanning 139 years, with the majority of samples within a 16‐year period (2006–2022). Overall, there were signatures of stability in haplotype diversity across the dominant 16‐year sampled period and across the additional historical specimens; however, the sample size was very limited, which may have contributed to this result (Table 2, Figure 3). Despite this limited sampling, four previously undetected haplotypes were identified from individual historical specimens for the CO1 marker, all of which were absent in contemporary samples (Figures 2 and 3, Table 2). Notably, one unique haplotype (C13) was recovered from a specimen collected in 1883 from Geraldton, a population that is now extinct. Additional unique haplotypes were observed in a 2008 specimen from the extinct Jurien Bay population (C14), as well as individuals from Marmion (2006; C15) and Hamelin Bay (2008; C16: Figures 2 and 3).

**TABLE 2:** Diversity metrics for *Scytothalia dorycarpa* specimens at six locations in Western Australia for multiple years for the cytochrome c oxidase subunit‐I (CO1) mitochondrial gene region and RuBisCO large chloroplast subunit (*rbc*L) gene region.

Marker	Population	Year	*n*	*K*	*S*	*P*_i_	*H*	*H*_d_
CO1	Geraldton*	1883	1	–	0	–	1	–
		1960	1	–	0	–	1	–
	Jurien Bay*	2008	3	–	2	–	2	–
	Lancelin	2022	14	0.429	3	0.001	4	0.396
	Marmion	~1800s	1	–	0	–	1	–
		2006	1	–	0	–	1	–
		2022	10	0.200	1	0.000	2	0.200
	Canal Rocks	2022	13	0.821	3	0.001	3	0.564
	Hamelin Bay	2008	6	0.677	2	0.001	3	0.600
		2010	5	0.400	1	0.001	2	0.400
		2022	12	0.470	2	0.001	3	0.439
*rbc*L	Jurien Bay*	2008	7	0.000	0	0.000	1	0.000
	Lancelin	2022	18	0.556	5	0.001	4	0.314
	Marmion	2006	3	–	0	–	1	–
		2022	13	0.000	0	0.000	1	0.000
	Canal Rocks	2022	15	0.267	2	0.000	2	0.133
	Hamelin Bay	2008	11	0.364	2	0.000	2	0.182
		2010	19	0.503	3	0.001	3	0.205
		2022	15	0.629	3	0.001	3	0.257

*Note*: Diversity metrics absent for locations with less than three samples present in a year, with * denoting population now extinct.

Abbreviations: *H*, number of haplotypes; *H*_d_, haplotype diversity; *K*, average number of nucleotide differences; *n*, sample size of DNA sequences in population set; *P*_i_, nucleotide diversity; S, number of segregating sites.

In contrast, no new haplotypes were identified for the *rbc*L gene marker from historical specimens (Figures 2 and 3, Table 2). The extinct population of Jurien Bay was dominated by the single widespread haplotype (R1) for the *rbc*L gene marker, which was shared across all historical specimens except those from Marmion collected in 2006, which was dominated by a single haplotype (R5) that was shared with Canal Rocks (2022) and Hamelin Bay (2008, 2010, and 2022; Figure 3). Haplotype diversity and identity were consistent in Hamelin Bay from 2008 to 2022 for the *rbc*L gene marker (Table 2, Figure 3). For the CO1 gene marker, there was a decrease in nucleotide and haplotype diversity across 2 years from 2008 and 2010, which corresponded in a shift in identity from C5 to C1 (Figure 3), but there was a subsequent increase in diversity 12 years later in 2022 for both metrics (Table 2). There was a notable change in haplotype identity at Marmion between 2006 and 2022, with the R5 haplotype observed in 2006 absent in 2022 (Figure 3).

## DISCUSSION

We identified spatial and temporal variation in haplotype diversity and identity in the climate threatened fucoid, *Scytothalia dorycarpa*, across an ~2700‐km portion of its geographic range. Overall, common haplotypes were broadly shared across the entire sampled area, indicating shared evolutionary histories. Our study leveraged historical specimens to reconstruct temporal trends, revealing stability in haplotype diversity over the 16‐year sampling period. Notably, populations at both the warm‐range edge and near the cool‐rear edge harbored unique haplotypes (some of which may now have been lost), while those in the Cape Naturaliste‐Leeuwin region (Western Australia) consistently exhibited high levels of diversity and unique haplotype identity, coinciding with stable temporal diversity and structure estimates. Critically, despite limited historical sample sizes, our results suggest that there is unique haplotype diversity present at fine scales along the sampled geographic range, with potential implications for the species' adaptive resilience.

### Contemporary spatial haplotype diversity and identity patterns revealed

Our results highlighted differences in haplotype composition and diversity among contemporary *Scytothalia dorycarpa* populations throughout the sampled range while also revealing that despite local differences, many haplotypes are broadly shared among populations. High haplotype diversity was concentrated in specific locations, notably in Hamelin Bay and Canal Rocks in the Cape Naturaliste–Leeuwin region across all markers and in the Bremer Bay population (~460 km east) for the CO1 marker. In contrast, several populations including the population at Port Elliot (~1700 km east of Esperance), which is the closest population we sampled to the leading edge of the species, exhibited low diversity but possessed a unique CO1 haplotype, highlighting both localized differentiation and broader regional ancestry. Ecological observations further indicate that populations differ in thermal tolerance across the range of the species, with these differences likely reflecting variation in localized haplotype diversity and adaptation (Bennett et al., 2015). Such contrasting patterns across populations suggest that present‐day diversity in *S. dorycarpa* reflects a legacy of historical processes, which may have been influenced by the contraction and expansion of species ranges during periods of sea level change in the Pleistocene (Hofreiter & Stewart, 2009) as well as population‐specific signatures of local adaptation, particularly at the warm‐range edge (Bennett et al., 2015). Similar patterns of connectivity and regional differentiation have been reported in other seaweeds, for which demographic shifts were influenced by past climatic oscillations and associated changes in habitat availability (Lee et al., 2012; Li et al., 2017; Luttikhuizen et al., 2018; Mueller et al., 2018; Ng et al., 2019; Nimbs et al., 2023). Although the specific evolutionary history of *S. dorycarpa* remains poorly resolved, other Fucalean species in Australia are believed to have evolved during a time of climatically stable conditions (Silberfeld et al., 2010, 2011), consistent with their relatively low tolerance to temperature fluctuations (Bennett et al., 2015). This interpretation is supported by the concentration of unique haplotypes in the Cape Naturaliste–Leeuwin region, which was consistent with persistence in local refugia, whereas the widespread occurrence of a few common haplotypes across distant sites suggests postglacial range expansion. Additionally, the spatial structuring of haplotype frequencies along the coast aligns with known fluctuations in connectivity driven by oceanographic and climatic variation, as discussed below.

Oceanographic features and life‐history traits may further explain the observed haplotype diversity and identity patterns in *Scytothalia*. The studied region is part of the Flindersian biogeographic province, influenced by the Leeuwin, Cresswell, and Flinders Currents, which vary seasonally in strength and direction (Akhir et al., 2020; Pearce & Pattiaratchi, 1999). The Leeuwin Current, a strong southward‐flowing boundary current, can facilitate alongshore transport of material and can potentially aid in the movement of detached thalli or reproductive fragments over considerable distances (Akhir et al., 2020; Pearce & Pattiaratchi, 1999). In contrast, the seasonally variable Cresswell and Flinders Currents flow northward along the southern coast and can promote localized retention or counter‐current dispersal (Bye, 1983; Middleton & Cirano, 2002). Such oceanographic dynamics provide the physical potential for connectivity across regions, yet their influence is constrained by the biology of the study species. *Scytothalia dorycarpa* is a Fucalean species, following a typical fucoid life cycle in which over 90% of propagules settle within meters of the parental thalli (Kendrick & Walker, 1995), and it lacks gas‐filled vesicles to aid rafting and, thus, gene flow. Previous studies suggested long‐distance dispersal of propagules as a means of gene flow and maintenance of genetic connectivity can be limited in Fucalean species (see Edgeloe et al., 2025), unless detached reproductive adult plants are distributed further afield, which is unlikely in this species (Schiel & Foster, 2006). Moreover, field and experimental evidence demonstrated that early life stages of *S. dorycarpa* are highly sensitive to elevated temperatures, with reduced recruitment and survival at warmer, low‐latitude reefs (Andrews et al., 2014), further constraining dispersal and reinforcing localized genetic structuring. Although regional currents could enhance the rare transport of propagules or thalli, the widespread haplotypes observed are more plausibly explained by rapid historical colonization and subsequent long‐term persistence rather than frequent, ongoing gene flow. The dominant haplotypes likely represent lineages that withstood repeated fluctuations in habitat and climate, while unique haplotypes in peripheral populations reflect isolation for considerable time, although under restricted dispersal.

### Rear and leading‐edge patterns of diversity and structure

Populations inhabiting the latitudinal margins of species ranges are critical for persistence, as they often contain unique adaptive or evolutionary genetic diversity that may be especially vulnerable to climate change (Assis et al., 2013; Coleman, Minne, et al., 2020; Minne et al., 2025; Nimbs et al., 2023; Vranken et al., 2021; Wood et al., 2021). In our study, we identified unique diversity and structure in the contemporary warm‐rear edge population of Lancelin across two gene markers (*rbc*L and CO1), with three private haplotypes detected for each marker and all also containing the common range‐wide haplotype across all markers. Although the Lancelin samples were collected as drift, the southward‐flowing Leeuwin Current and prevailing poleward winds suggest they may have originated from further north or are local (Akhir et al., 2020). We hypothesize that the Lancelin population is locally isolated, with the observed private haplotypes persisting through historical climate fluctuations, including the 2011 MHW. It is also possible that the contemporary drift samples could have originated from deeper environments offshore, representing either remnant local populations that are less exposed to surface warming or source populations from other regions carried by oceanic currents, highlighting the complexity of gene flow and the potential for cryptic refugia contributing to the persistence of unique haplotypes at the warm‐rear edge (Nimbs et al., 2023). Additionally, the now‐extinct northern populations of Geraldton and Jurien Bay were genetically similar to Lancelin in the CO1 gene marker, sharing the common haplotype (C1), but also contained private haplotypes not detected elsewhere (despite low sample sizes). The extinction of these populations, therefore, likely resulted in the loss of lineages that may have been adapted to warmer conditions at the rear edge of the species, representing an erosion of functional genetic diversity and adaptive potential. However, due to local adaptation, all populations may be equally vulnerable to extreme warming events (Bennett et al., 2015), underscoring the need to consider both local adaptation and rear‐edge effects in vulnerability assessments (i.e., Coleman et al., 2022).

At the opposite margin, the population of Port Elliot in South Australia was genetically distinct for the CO1 gene marker, containing a private haplotype (C9), whereas for *rbc*L, *trn*W‐1, and *cox*3, it comprised the range‐wide common haplotypes. The presence of the unique CO1 haplotype caused some of the Port Elliot samples to cluster separately on the phylogenetic tree, yet the occurrence of common haplotypes across the other markers points to shared ancestry across the Great Australian Bight, despite signals of potential localized differentiation or recent isolation at the range edge. However, given that Port Elliot is geographically distant from the other sampled populations, additional sampling across intermediate sites would be needed to determine whether its unique haplotype is a result of isolation by distance or other historical processes, rather than a result of adaptation to a distinct environment. Such unique haplotype diversity and identity at range edges is consistent with patterns in other seaweeds, including *Fucus ceranoides* (Neiva et al., 2012), *Ishige okamurae* (Lee et al., 2012), and *Saccharina japonica* (Zhang et al., 2019). Notably, the Laminarian *Ecklonia radiata* also harbored unique genetic diversity at its rear edges in eastern Australia (Nimbs et al., 2023), Western Australia, and South Africa (Coleman et al., 2022; Madeira et al., 2024), with SNP studies confirming similar contemporary patterns on both the east (Minne et al., 2025) and west (Vranken et al., 2021, 2025) Australian range edges. Likewise, *Nereia lophocladia* exhibited unique genetic diversity at its warm‐range edge in eastern Australia (Mamo et al., 2021). These results, particularly those from Western Australia, reinforce the view that warm edges are reservoirs of unique genetic diversity, likely shaped by historical bottlenecks or limited gene flow, but also highlight that ongoing and future range shifts risk the irreversible loss of this diversity. Although local adaptation remains a possible factor, further studies using nuclear markers or genome‐wide approaches would be needed to test for adaptive divergence.

### Unique genetic diversity in Cape Naturalist‐Leeuwin region

Across all markers, our findings consistently revealed the Cape Naturalist‐Leeuwin region, Canal Rocks, and Hamelin Bay had unique genetic structure and the highest haplotype diversity out of all sampled locations. Haplotype structure in the Cape Naturalist‐Leeuwin region was particularly distinctive, with private haplotypes unique to both locations that were not observed in locations further north or east, yet the common haplotypes were shared with surrounding locations ~300 km away. Notably, samples from the Cape Naturalist‐Leeuwin region that had unique haplotypes formed their own cluster on the phylogenetic tree. The Cape Naturalist‐Leeuwin region is characterized by unique oceanographic conditions, with two major currents influencing the region: (1) Leeuwin Current and (2) Capes Current (Akhir et al., 2020), with the equatorward‐flowing Capes current developing in the inner and mid‐shelf in the summer months, creating upwelling of cooler continental shelf waters between Cape Leeuwin and Cape Naturaliste (Gersbach et al., 1999; Pearce & Pattiaratchi, 1999). It is probable that a combination of offshore upwelling and historical sea‐level changes have created physical barriers for gene flow between the Cape Naturalist‐Leeuwin region and surrounding areas, inherently leading to localized haplotypes that are private to the region. Interestingly, our results for the high genetic diversity and structure in the Cape Naturalist‐Leeuwin region are not unique, with similar findings observed for other marine taxa including the seagrass *Posidonia australis* (Sinclair et al., 2023) and seaweed species *Ecklonia radiata* (Vranken et al., 2021) and *Cystophora racemosa* (Edgeloe et al., 2025). Given the unique genetic diversity of the Cape Naturalist‐Leeuwin region, targeted conservation efforts are critical, to safeguard its high genetic diversity as well as its role as a potential source of alleles for recolonizing populations further north. Conservation strategies should prioritize preserving these genetic hotspots to maintain the adaptive potential of the species under future environmental change, including methods such as spore or tissue banking to safeguard rare lineages.

### Historical patterns of genetic diversity

Although we were unable to successfully amplify markers from many historical and herbarium samples, we were able to identify unique historical haplotype diversity and identity across multiple timepoints, particularly in the Cape Naturalist‐Leeuwin region in the Hamelin Bay population. Haplotype diversity and identity remained almost unchanged between 2008 and 2022 at Hamelin Bay for the *rbc*L gene marker, with only one additional haplotype being observed in 2022 that was absent in all other years. It is possible that the newly observed haplotype was a warm‐affinity genotype that persisted through the 2011 MHW but was not revealed in other sampling years due to low sample sizes. Notably, this result differed from Gurgel et al. (2020) for the *rcb*L marker, which showed a 28% decline in *rbc*L haplotype diversity between 2008 and 2012 at Hamelin Bay. The discrepancy of these results may be underpinned by sample size, as we used fewer samples per site than Gurgel et al. (2020). Further, despite low sample size (2008, *n* = 6 and 2010, *n* = 5), we were able to detect a change in CO1 nucleotide and haplotype diversity from 2008 to 2010, which is consistent with the findings of Gurgel et al. (2020) for the CO1 marker. Interestingly, our longer time series also revealed a subsequent increase in diversity in 2022 for both metrics, which may indicate that haplotypes lost during or after the 2011 MHW may be recovering or that novel mutations have subsequently emerged as a result of this severe event. Considering the life history of *Scytothalia dorycarpa* (Andrews et al., 2014), which assumes a generation time of between 2 and 5 years, the temporal span of our sampling likely encompasses a few generations, suggesting that genetic drift alone would have limited impact on haplotype frequencies over this interval. Therefore, observed changes are more likely to reflect recovery following the MHW, novel mutations, or gene flow rather than genetic drift. This warrants further investigation using a higher sample size across future timepoints to clarify the role of adaptation to changing environmental conditions (e.g., warming sea temperatures) and/or gene flow are contributing to the higher diversity observed.

### Future directions and conclusions

Our study provides a critical step in establishing baseline haplotype diversity estimates for *Scytothalia dorycarpa* across its range. Prior to this study, minimal genetic knowledge existed for *S. dorycarpa*, with the exception of one molecular study which explored only two of our sampled locations (Gurgel et al., 2020). Importantly, a next step is to explore molecular markers that represent the nuclear genome (rather than organellar genomes) such as exploration of single nucleotide polymorphisms via next‐generation sequencing, which could show potential signatures of selection and adaptation. Future studies would benefit from additional replication to verify haplotypes, particularly for historical herbarium samples where DNA was degraded and replicate amplifications may be challenging. Additionally, we recommend further genetic sampling over a larger portion of the geographic range between Esperance and Port Elliot to develop an understanding of the degree of genetic connectivity between the cool near‐leading edge population at Port Elliot and the rest of the range of the species. The findings of our study have strong implications for the management of *S. dorycarpa* populations, particularly at the warm‐range edge Lancelin and in the Cape Naturalist‐Leeuwin region—Canal Rocks and Hamelin Bay. We recommend a focus on conservation of these locations, specifically due to the unique diversity and identity exhibited, which can possibly be linked to locally adapted genotypes and/or historical refugia. These insights emphasize the importance of implementing localized conservation and management strategies that priorities the preservation of genetic diversity across populations, particularly at the warm‐rear and cool near‐leading edges, as well as historically isolated refugial areas, to ensure the long‐term resilience of *S. dorycarpa* in the face of climate change.

## AUTHOR CONTRIBUTIONS

**Jane M. Edgeloe:** Conceptualization (equal); data curation (lead); formal analysis (lead); funding acquisition (supporting); investigation (lead); methodology (lead); project administration (lead); resources (lead); software (lead); validation (lead); visualization (lead); writing – original draft (lead); writing – review and editing (lead). **Melinda A. Coleman:** Conceptualization (supporting); funding acquisition (supporting); investigation (supporting); methodology (supporting); project administration (supporting); supervision (supporting); writing – review and editing (supporting). **Georgina Wood:** Conceptualization (supporting); data curation (supporting); formal analysis (supporting); investigation (supporting); methodology (supporting); supervision (supporting); writing – review and editing (supporting). **Samuel Starko:** Conceptualization (supporting); data curation (supporting); formal analysis (supporting); investigation (supporting); methodology (supporting); supervision (supporting); writing – review and editing (supporting). **Matt J. Nimbs:** Formal analysis (supporting); investigation (supporting); methodology (supporting); visualization (supporting); writing – review and editing (supporting). **Jacqueline Batley:** Methodology (supporting); resources (supporting); supervision (supporting); writing – review and editing (supporting). **Thomas Wernberg:** Conceptualization (supporting); funding acquisition (lead); investigation (supporting); methodology (supporting); project administration (supporting); resources (supporting); supervision (supporting); writing – review and editing (supporting).

## Supporting information


**Table S1.** Key resources information.
**Table S2.** Sampling information of historical *Scytothalia dorycarpa* specimens attained for this study.
**Figure S1.** Phylogenetic reconstruction for *Scytothalia dorycarpa* based on concatenated *rbc*L, CO1, *cox*3 and *trn*W‐1 sequences using a maximum likelihood (ML) tree building algorithm.

## Data Availability

Genetic data are available on FigShare: 10.6084/m9.figshare.30154462 and GenBANK (PX548838 – PX548865).
