# Supplementary material for: Temporal stability and spatial patterns of genetic diversity in populations of the climate‐vulnerable fucoid Scytothalia dorycarpa
Source: J Phycol. 2026 Mar 24;62(2):715–31. doi: 10.1111/jpy.70156 (PMC13103687; doi:10.1111/jpy.70156)
Supplement: Supplementary file 1 — Table S1. Key resources information. Table S2. Sampling information of historical Scytothalia dorycarpa specimens attained for this study. Figure S1. Phylogenetic reconstruction for Scytothalia dorycarpa based on concatenated rbcL, CO1, cox3 and trnW‐1 sequences using a maximum likelihood (ML) tree building algorithm. [file JPY-62-715-s001.docx]

**Supplemental Information for:**

**Temporal stability and spatial patterns of genetic diversity in populations of the climate-vulnerable fucoid *Scytothalia dorycarpa***

**Jane M. Edgeloe, Melinda A. Coleman, Georgina Wood, Samuel Starko, Matt J. Nimbs, Jacqueline Batley and Thomas Wernberg**

**Table of Contents:**

| **Table S1:** Key resources information | Page 2 |
| --- | --- |
| **Table S2:** Sampling information of historical *Scytothalia dorycarpa* specimens attained for this study. | Page 4 |
| **Figure S1:** Phylogenetic reconstruction for *Scytothalia dorycarpa* based on concatenated *rbc*L, CO1*, cox*3 and *trn*W*-*1 sequences using a maximum likelihood (ML) tree building algorithm. Values provided as approximate Bayesian support (right) and ML bootstrap (left), with the middle value the posterior probability from Bayesian analysis. | Page 5 |

**Table S1:** Key resources information

|  | **REAGENT or RESOURCE** |  |  | **Source** |
| --- | --- | --- | --- | --- |
| **Gene region** | **Oligonucleotides** | **Annealing temperature/ Amplicon size** | **Position of primer within gene bp (gene accession for comparison in brackets)** |  |
| RuBisCO large subunit (*rbc*L) chloroplast marker | **KL2** (GAT GCT GAT TAT AAC GTT AAAG) | 56.3°C /  1092 bp | 88-132 (MG922855.1) | Lane et al., 2007 |
|  | **KL8** (GTT GGT GCA TTT GAC CACA) |  | 1166-1180 (MG922855.1) | Lane et al., 2007 |
| partial mitochondrial *cox*1-5’ barcoding region (CO1) | **Gaz F1** (TCA ACA AAT CAT AAA GAT ATT GG) | 56.2°C /  710 bp | 97-118 (MG922856.1) | Saunders. 2005 |
|  | **Gaz R1_new** (ACT TCW GGA TGT CCA AAA AAY CA) |  | 784-807 (MG922856.1) | This paper |
| partial cytochrome c oxidase subunit-3 (*cox*3) mitochondrial gene region | **Inside_F (**GGA AGC CTT GTT TGA GGG AC) | 57.3°C /  250 bp | 273-293 (MG922856.1) | This paper |
|  | **Inside_R (**TCA AAA GTG GCA ACC CCC AA) |  | 503-523 (MG922856.1) | This paper |
| partial mitochondrial intergenic spacer region (*trn*W-1) | **trnW-1-F** (GGG GTT CAA ATC CCT CTC TT) | 61.7°C /  190 bp | - | Voisin et al., 2005 |
|  | **trnW-1-R** (CCT ACA TTG TTA GCT TCA TGA GAA) |  | - | Voisin et al.*,* 2005 |
| RuBisCO large subunit (*rbc*L) chloroplast marker  (Historical) | **KL2** (GAT GCT GAT TAT AAC GTT AAAG) | 52.8°C /  391 bp | 88-132 (MG922855.1) | Lane et al., 2007 |
|  | **KL2_R1** (TGT AGC AGG ACC TTG AAA AGT CT) |  | 456-479 (MG922855.1) | This paper |
|  | **KL2_F2** (TTG GTA ATG TAT TTG GTT TTA AAG CAG) | 57.4°C /  491 bp | 381-407 (MG922855.1) | This paper |
|  | **KL2­_R2** (TCG TGC CCA AA TAG CCA TCG) |  | 853-872 (MG922855.1) | This paper |
|  | **KL2_F3** (ATC TTA ATG TTA CTG CAG CAA CAA) | 55.9°C /  445 bp | 735-758 (MG922855.1) | This paper |
|  | **KL8** (GTT GGT GCA TTT GAC CACA) |  | 1166-1180 (MG922855.1) | Lane et al., 2007 |
| partial mitochondrial *cox*1-5’ barcoding region (CO1)  (Historical) | **Gaz F1** (TCA ACA AAT CAT AAA GAT ATT GG) | 55.3°C /  261 bp | 97-118 (MG922856.1) | Saunders. 2005 |
|  | **CO1_R1** (CRC CAT ATY AGG AGC ACC AA) |  | 340-358 (MG922856.1) | This paper |
|  | **CO1_F2** (TGC CCR TAC KTA TAG GTR GTT T) | 55.3°C /  313 bp | 292-312 (MG922856.1) | This paper |
|  | **CO1_R2** (ACC AGG AGC ACR CAT GTT AA) |  | 586-605 (MG922856.1) | This paper |
|  | **CO1_F3** (GGT GCG GCC TCT ATC TTR GG) | 61.4°C /  267 bp | 540-559 (MG922856.1) | This paper |
|  | **Gaz R1_new** (ACT TCW GGA TGT CCA AAA AAY CA) |  | 784-807 (MG922856.1) | This paper |

**References (Table S1)**

Lane, C. E., Lindstrom, S. C., & Saunders, G. W. (2007). A molecular assessment of northeast Pacific Alaria species (Laminariales, Phaeophyceae) with reference to the utility of DNA barcoding. *Molecular phylogenetics and evolution*, *44*(2), 634-648. https://doi.org/10.1016/j.ympev.2007.03.016

Saunders, G. W. (2005). Applying DNA barcoding to red macroalgae: a preliminary appraisal holds promise for future applications. *Philosophical Transactions of the Royal Society of London. Series B. Biological Sciences*, *360*(1462), 1879–1888. https://doi.org/10.1098/rstb.2005.1719

Voisin, M., Engel, C. R., & Viard, F. (2005). Differential shuffling of native genetic diversity across introduced regions in a brown alga: aquaculture vs. maritime traffic effects. *Proceedings of the National Academy of Sciences - PNAS*, *102*(15), 5432-5437. https://doi.org/10.1073/pnas.0501754102

**Table S2:** Sampling information of historical Scytothalia dorycarpa specimens attained for this study.

| **Location** | **Number samples** | **Year** | **Latitude** | **Longitude** | **GB accession** | **Source** | **Extinct** |
| --- | --- | --- | --- | --- | --- | --- | --- |
| Albany | 1 | 1881 | -35.0000 | 117.8667 | MEL 0688309A | Melbourne |  |
| Geraldton | 1 | 1898 | -28.7667 | 114.6167 | MEL 2293533A | Melbourne | Y |
| Cottesloe | 1 | 1899 | -32.0000 | 115.7500 | MEL 2293536A | Melbourne |  |
| North Beach | 1 | 1931 | -31.8572 | 115.7504 | AD-A 02035 | Adelaide |  |
| Dongara, Irwin River mouth | 1 | 1948 | -29.2508 | 114.9306 | AD-A 55970 | Adelaide | Y |
| Greenough River, S of Geraldton | 1 | 1948 | -28.8653 | 114.635 | AD-A 55968 | Adelaide | Y |
| Bluff Point, Geraldton | 1 | 1954 | -28.7358 | 114.6025 | PERTH 4153081 | Perth | Y |
| Dongara | 1 | 1960 | -29.2508 | 114.9306 | AD-A 24550 | Adelaide | Y |
| Cliff Head | 1 | 1979 | -29.5333 | 114.9833 | PERTH 4153111 | Perth | Y |
| Cliff Head | 1 | 1979 | -29.5167 | 114.9833 | PERTH 7169140 | Perth | Y |
| Cliff Head | 1 | 1979 | -29.5208 | 114.9948 | AD-A 51218 | Adelaide | Y |
| Jurien Bay | 1 | 2005 | -30.3393 | 114.9798 | PERTH 7391250 | Perth | Y |
| Marmion | 3 | 2006 | -31.7779 | 115.6761 |  | T. Wernberg |  |
| Jurien Bay | 8 | 2008 | -30.2659 | 114.9754 |  | T. Wernberg | Y |
| Hamelin Bay | 15 | 2008 | -34.2682 | 115.0196 |  | T. Wernberg |  |
| Hamelin Bay | 20 | 2010 | -34.2562 | 115.0009 |  | T. Wernberg |  |
| King George Sound, Albany | 1 | n.d | -35.0000 | 117.9667 | MEL 0688330A | Melbourne |  |
| Champion Bay, Geraldton | 1 | n.d | -28.7667 | 114.6333 | MEL 0688315A | Melbourne | Y |
| Champion Bay, Geraldton | 1 | n.d | -28.7667 | 114.6333 | MEL 0688314A | Melbourne | Y |
| City Beach | 1 | n.d | -31.9333 | 115.7617 | PERTH 4153073 | Perth |  |
| Dongara | 1 | n.d | -29.2500 | 114.9167 | PERTH 6996132 | Perth | Y |
| Nanarup, Albany | 1 | n.d | -34.9833 | 118.0500 | PERTH 7040733 | Perth |  |
| North Beach | 1 | n.d | -31.8500 | 115.7500 | PERTH 6996167 | Perth |  |

Note: GB accession = herbarium accession ID. Source = herbarium storage location.


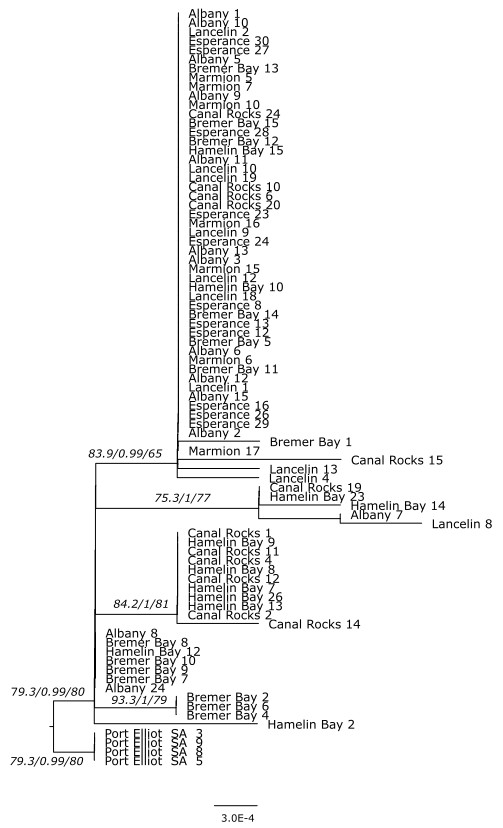


**Figure S1:** Phylogenetic reconstruction for Scytothalia dorycarpa based on concatenated rbcL, CO1, cox3 and trnW-1 sequences using a maximum likelihood (ML) tree building algorithm. Values provided as approximate Bayesian support (right) and ML bootstrap (left), with the middle value the posterior probability from Bayesian analysis.
